# Baseline prevalence of lung cancer screening and associated factors among women in breast cancer screening clinics prior to the Pink & Pearl program: A retrospective analysis of electronic health records

**DOI:** 10.21203/rs.3.rs-10998785/v1

**Published:** 2026-10-06

**Authors:** Beryne Odeny, Sridharan Gopalsamy Ramaswamy, Benjamin Bowe, Eyerusalem Zewde, Aren Mnatzakanian, Busayo Akinloye, Thomas Odeny, Michelle V. Lee

**Affiliations:** 1 School of Medicine, Department of Surgery, WashU, St. Louis, MO, USA; 2 Department of Medical Imaging, University of Toronto, Ontario, Canada; 3 School of Medicine, Department of Medicine, WashU, St. Louis, MO, USA; 4 School of Medicine, Department of Radiology, WashU, St. Louis, MO, USA

**Keywords:** lung cancer screening, breast cancer screening, cancer screening disparities, electronic health records, smoking history documentation, low-dose computed tomography

## Abstract

**Introduction:**

Lung cancer screening (LCS) remains underutilized, with 18% uptake nationally, despite established evidence-based guidelines, particularly among women who engage with preventive care through breast cancer screening (BCS). We characterized baseline LCS uptake and its correlates among women receiving BCS at two participating hospitals.

**Methods:**

We conducted a retrospective cohort study using six years of electronic health record data (October 2019–November 2025) from Barnes-Jewish West County Hospital and Christian Hospital. Women aged 50–80 with any documented smoking history were included. The primary outcome was LCS uptake, defined as evidence of at least one low-dose computed tomography scan. We conducted multivariable logistic regression models and assessed for effect modification by race and area deprivation using likelihood ratio tests.

**Results:**

Of 9,686 women with smoking history, 3,209 (33.1%) had received at least one LCS. Pack-year documentation status (AOR 2.90; 95% CI: 2.63, 3.19), tobacco cessation medication use (AOR 2.21; 95% CI: 1.98, 2.48), and current smoking (AOR 1.84; 95% CI: 1.64, 2.07) were the factors most strongly associated with uptake. Non-Hispanic Black women had lower adjusted odds of obtaining LCS compared to non-Hispanic White women (AOR 0.87; 95% CI: 0.78, 0.98); estimates in the smaller pack-year subgroups were similar in direction but imprecise. Alcohol use was associated with lower odds of LCS uptake (0.81, 95% CI: 0.73, 0.89). Effect modification by race and area deprivation were not significant.

**Conclusions:**

Baseline LCS uptake exceeded the national average; however, racial disparities were observed. Completeness of smoking history documentation, tobacco cessation medication use, and current smoking status were strongly associated with LCS uptake. These findings highlight the need for strategies to improve smoking history documentation and promote tobacco cessation efforts in cancer screening programs and establish the baseline against which the Pink & Pearl program, a co-located breast and lung cancer screening initiative at these sites, will be evaluated.

## INTRODUCTION

Lung cancer remains the leading cause of cancer-related mortality in the U.S., despite substantial progress in the prevention, detection, and treatment of other cancer types.^1^ In 2026, an estimated 229,410 new cases and 124,990 deaths are expected in the U.S., with a 5-year relative survival rate of ~27% overall.^2,3^ Poor survival outcomes are partly because lung cancer is often diagnosed at a late stage when treatment is less effective.^1^

Five-year survival is several times higher when lung cancer is diagnosed at a localized rather than a distant stage, which is the rationale for screening asymptomatic high-risk adults.^1^ Low-dose computed tomography (LDCT) screening has reduced lung cancer mortality.^4,5^ The National Lung Screening Trial reported a 20% relative reduction compared to chest radiography.^4^ These findings were confirmed with the NELSON trial, indicating significant mortality reductions of ~24% in men over a 10-year period, with evidence suggesting a potentially greater benefit in women.^5^ Despite the existence of evidence-based guidelines and concerted implementation efforts, uptake of lung cancer screening (LCS) remains low.^6^ Among the 8.5 million current and former smokers eligible for LCS, a mere 18% have undergone screening,^6^ and adherence to annual re-screening is similarly low, at 37–51%.^7^

Missouri, and specifically the St. Louis metropolitan region, provides a relevant context to examine these issues. Populations served by a single health system in this region differ substantially in neighborhood socioeconomic deprivation, insurance coverage, and smoking prevalence, and screening programs may reach them unevenly. Describing who does and does not receive LCS within such a system is a necessary first step toward tailoring screening delivery for greater impact.

Women have lower LCS uptake than men despite higher engagement with other cancer screening, potentially explained by sex-specific patterns in smoking and cessation behaviors.^8,9^ Women report higher instances of light and nondaily/intermittent smoking, which may diminish risk perception and decrease engagement with preventative services.^10^ Furthermore, stigma associated with smoking and lung disease serves as a barrier to seeking LCS, and women report lower awareness of LCS than men.^8,11^ Thus, women presenting for BCS represent a practical opportunity to address these gaps: they are already engaged with preventive care and in routine contact with a health system that could identify LCS eligibility, yet their LCS uptake in this setting remains poorly described.

We conducted a six-year retrospective analysis of electronic health record (EHR) data from Barnes-Jewish West County Hospital (BJWCH) and Christian Hospital in St. Louis to characterize LCS uptake among women receiving breast cancer screening (BCS). This analysis establishes a baseline prior to implementation of the Pink & Pearl program, a co-located BCS and LCS initiative at these sites. Specifically, we aim to: 1) determine the baseline prevalence of LCS uptake among women aged 50–80 with a smoking history who are receiving BCS; 2) compare LCS uptake across USPSTF eligibility subgroups defined by smoking documentation; and 3) identify patient-level factors associated with LCS uptake. We hypothesized that LCS uptake in this BCS-engaged population would exceed national estimates, that uptake would be lower among women with incomplete smoking history documentation, and that uptake would differ by race and neighborhood deprivation.

## METHODS

### Study Design and Data sources

We performed a retrospective cohort study using electronic health record (EHR) data from the Barnes-Jewish Healthcare (BJC) system in Saint Louis, Missouri. Specifically, data were extracted from the EPIC EHR system for women who participated in BCS services at Barnes-Jewish West County Hospital (BJWCH) and Christian Hospital (CH), spanning a six-year period from October 2019 through November 2025. We extracted data for women aged 50 – 80 with a documented history of tobacco smoking, whether current or former, who had undergone at least one mammography visit during the study period. We extracted data on pack years of smoking, when it was documented, and whether individuals had any history of LCS through November 2025. Cohort selection is summarized in Figure 1.

### Population

The BCS service at BJWCH predominantly serves a higher-income, primarily White, insured population, with ~2,500 women screened annually in West County area, where smoking rates are <10%. In contrast, Christian Hospital operates a high-risk breast cancer clinic that annually serves >9,000 women, primarily from low-income, uninsured and racially diverse backgrounds, with a significant number being Black women, and smoking rates ranging from 15% to 20%.^12^ Both hospitals are integral components of the Barnes-Jewish Healthcare system, which collaborates with the Siteman Cancer Center (SCC), a National Cancer Institute-designated comprehensive cancer center. SCC serves a diverse population from the St. Louis metropolitan region, encompassing 82 counties in eastern Missouri and southern Illinois.^13^ SCC’s catchment area is 79.5% non-Hispanic White, followed by 13.7% Black, 2% Asian, and 4.8% representing other racial categories. Around 3% of the population identifies as Hispanic/Latino. SCC is instrumental in meeting healthcare needs across a broad socioeconomic range, including medically underserved areas, which account for 29% of the population, and rural zip codes accounting for 15% of the population.^12^

### Data Elements

Our dataset includes a comprehensive range of variables: patient demographics; smoking history encompassing smoking status, pack-years, years since cessation, and tobacco cessation medication use; preventive care use (e.g., BCS and LCS history); insurance status; clinical history (covering any history of cancer or lung disease); place based characteristics; and social determinants of health data. Regarding data on race and ethnicity, the EHR typically includes a field for recording the patient’s race and ethnicity, which is usually self-reported during registration. Race and ethnicity categories in our dataset include non-Hispanic White, non-Hispanic Black, Asian, Hispanic, and Other.

### LCS Eligibility

The 2021 USPSTF guidelines define LCS eligibility as adults aged 50–80 with a smoking history of at least 20 pack-years who currently smoke or quit within the past 15 years. Because pack-years and quit dates were frequently undocumented in this dataset, strict application of these criteria was not possible, and we therefore defined subgroups by documentation status as described below. Strict application of USPSTF criteria would exclude most women whose eligibility could not be determined due to incomplete documentation rather than absence of risk. We therefore defined our analytic cohort using broader criteria: women aged 50–80 with any documented smoking history (current or former smoker), regardless of whether pack-years or quit date were recorded. This approach allowed us to characterize the full population of women with smoking exposure in the BCS setting. To examine LCS uptake among women who met strict USPSTF thresholds, we conducted subgroup analyses restricted to those with documented pack-years of 20 or more (Tables 3 and 4). We also created a binary indicator for pack-year documentation status (documented vs. missing) and included it as a covariate in the whole-cohort regression model to account for the association between clinical documentation completeness and LCS uptake.

To characterize socioeconomic disadvantage, we used Area Deprivation Index (ADI) scores via the Center for Health Disparities Research data last updated in 2020. ADI is a validated metric originally developed by the Health Resources & Services Administration and ranks neighborhoods at the Census block group level based on income, education, employment, and housing conditions. We calculated mean ADI national percentile ranks at the ZIP code level and grouped neighborhoods into four ordered categories of socioeconomic deprivation: 1 (least deprived), 2, 3, and 4 (most deprived). Categories were defined by fixed cutpoints in the national percentile distribution and are therefore unevenly distributed within our cohort, which is concentrated in more deprived neighborhoods.

### Outcomes

Our primary outcome was LCS uptake, defined as evidence of at least one LDCT scan for LCS purposes in the EHR. We assessed this outcome across the full analytic cohort of women with any documented smoking history and within two subgroups defined by increasing specificity of USPSTF eligibility criteria. Because the 20 pack-year threshold applies to current and former smokers alike, the first subgroup comprised current smokers with documented pack-years of 20 or more, and the second comprised former smokers meeting the same pack-year threshold who additionally had a recorded quit date, as quit date documentation is required to determine whether cessation occurred within the past 15 years. We examined variability in LCS uptake across demographic, socioeconomic, clinical, and place-based characteristics within each subgroup.

### Statistical analysis

We summarized demographic, smoking, screening, clinical, and place-based characteristics of the analytic cohort by LCS status using medians with interquartile ranges for continuous variables and frequencies with percentages for categorical variables (Table 1). Unadjusted odds ratios (OR) with 95% confidence intervals were derived through simple logistic regression for each covariate. We then performed multivariable logistic regression to identify factors independently associated with LCS uptake, adjusting for age group, race/ethnicity, insurance type, ADI category, smoking status, pack-year documentation status, tobacco cessation medication use, cancer history, hospital site, alcohol use, and number of BCS encounters. Eight women with Medicaid-only coverage were excluded from regression models due to unstable parameter estimates. In the pack-year subgroup models (Tables 3 and 4), the binary pack-year documentation indicator was removed as a covariate because it had no variation within these subgroups, where all women had documented pack-years. Race/ethnicity categories were collapsed in smaller subgroup models where sparse data or zero cells prevented model convergence: Asian, Hispanic, and Other were combined in Table 3 (current smokers). In Table 4 (former smokers), race was retained as three categories (non-Hispanic White, non-Hispanic Black, and Other) and complete separation in the Other category was addressed through penalized estimation rather than collapsing. Statistical significance was assessed at a two-sided alpha level of 0.05. All analyses were conducted using R version 4.5.2 (R Foundation for Statistical Computing, Vienna, Austria).

Effect modification by race and area deprivation (ADI) was assessed using interaction terms and likelihood ratio tests comparing main-effects models to models with the corresponding interaction term in the whole-cohort analysis (Table 2). Interaction testing was not performed in the current-smoker and former-smoker subgroups (Tables 3 and 4) due to limited sample size. In Table 4, the Other race category (n=6) exhibited complete separation with no women in the unscreened cell; we therefore fit Table 4 using Firth penalized logistic regression with profile likelihood 95% confidence intervals.

### Handling missing data

Five variables (education, marital status, food insecurity score, financial insecurity score, and medical transportation needs) were excluded from analysis due to missing rates exceeding 94%. Pack-year data were missing for 63% of the analytic cohort, and quit status was undocumented for 79%. Rather than impute these values, we retained pack-year documentation status as a binary covariate in the whole-cohort model and restricted subgroup analyses to women with complete data on the relevant smoking variables. Cessation medication status was derived from pharmacy records, with the absence of a prescription record treated as no cessation medication use. This assumption may misclassify women who obtained cessation medication outside the health system. Descriptive statistics excluded missing observations from percentage calculations for each variable.

### Ethical approval

The Institutional Review Board of Washington University approved this study and granted a waiver of informed consent for accessing protected health information within the BJC health record (EHR) database. After data extraction from the Epic EHR system, direct identifiers were removed prior to analysis. Residential ZIP code was retained solely to link participants to Area Deprivation Index values and was not used in any other analysis or reported at the ZIP code level.

## Results

### Population Characteristics

The Epic extract identified 9,747 women aged 50 to 80 with a mammogram and documented smoking status at the two study sites between October 2019 and November 2025. Of these, 61 did not meet study inclusion criteria and were excluded, yielding an analytic cohort of 9,686 women. An additional 8 women with Medicaid as their only insurance were excluded from regression models because of unstable parameter estimates, leaving 9,678 women in adjusted analyses.

Of the 9,686 women in the analytic cohort, 3,209 (33.1%) had evidence of at least one LCS (Table 1). The median age at most recent encounter was 66 years (IQR: 59, 72). Based on age at cohort entry, 72% of women were under 65 and 28% were 65 or older. The cohort was roughly evenly split by race: 4,818 women (50%) were non-Hispanic Black and 4,625 (48%) were non-Hispanic White. The majority resided in the most deprived ADI category (64%), received care at Christian Hospital (73%), and were former smokers (77%). Pack-year data were documented for 37% of the full analytic cohort, and quit status was documented for 21% of former smokers. Documentation patterns differed by LCS status: 57% of women with LCS had recorded pack-years, compared to 27% of those without. Tobacco cessation medication use was also more prevalent among screened women (38% vs. 17%).

### Factors Associated with LCS Uptake in the Whole Cohort

In the whole-cohort regression model (N = 9,678; Table 2), the factor most strongly associated with LCS uptake was pack-year documentation status. Women with documented pack-years had nearly three times the odds of having received LCS compared to those without (AOR 2.90; 95% CI: 2.63, 3.19). Tobacco cessation medication use (AOR 2.21; 95% CI: 1.98, 2.48) and current smoking status (AOR 1.84; 95% CI: 1.64, 2.07) were also strongly associated with LCS uptake. Women aged 65 or older had higher odds of LCS than younger women (AOR 1.45; 95% CI: 1.30, 1.62), as did those with Medicare (AOR 1.50; 95% CI: 1.33, 1.70) or multiple insurance coverage (AOR 1.44; 95% CI: 1.27, 1.64) relative to private insurance. Women residing in the most deprived ADI category had higher adjusted odds of LCS uptake than those in the least deprived category (AOR 1.43; 95% CI: 1.14, 1.81). Non-Hispanic Black women had modestly lower odds of uptake compared to non-Hispanic White women after adjustment (AOR 0.87; 95% CI: 0.78, 0.98). Cancer history was positively associated with uptake (AOR 1.61; 95% CI: 1.45, 1.78), while alcohol use was inversely associated (AOR 0.81; 95% CI: 0.73, 0.89). Tests for effect modification of race by hospital site (likelihood ratio p = 0.26) and by ADI category (p = 0.88) were not statistically significant.

### Current and Former Smoker Subgroups

Among current smokers with pack-years of 20 or more (N = 627; Table 3), uptake reached 78.3%. Cessation medication use (AOR 1.74; 95% CI: 1.15, 2.65) and BCS encounters (AOR 1.30; 95% CI: 1.18, 1.43) were the only factors significantly associated with uptake in adjusted models. The adjusted odds of LCS uptake were lower for non-Hispanic Black than non-Hispanic White women (AOR 0.65; 95% CI: 0.40, 1.03), though the confidence interval crossed the null. Race categories were collapsed in this model due to small cell sizes in the Asian, Hispanic, and other groups.

When the analysis was further restricted to former smokers with pack-years of 20 or more and a documented quit date (N = 394; Table 4), uptake was 86.8%. Christian Hospital site was the only factor significantly associated with LCS in adjusted analyses (AOR 2.90; 95% CI: 1.23, 6.79). The adjusted odds were similarly lower for non-Hispanic Black than non-Hispanic White women (AOR 0.77; 95% CI: 0.38, 1.52), and the estimate for the remaining racial categories was imprecise, reflecting the small subgroup size. Confidence intervals were wide across most covariates, consistent with the reduced sample size.

## RESULTS TABLES

Baseline LCS Prevalence and Correlates Among Women in Breast Cancer Screening

## DISCUSSION

In this six-year retrospective cohort of 9,686 women receiving BCS at two Barnes-Jewish Healthcare hospitals, 33.1% received at least one LCS. Pack-year documentation status showed the largest adjusted odds ratio in the whole-cohort model (AOR 2.90), followed by tobacco cessation medication use (AOR 2.21) and current smoking status (AOR 1.84). Non-Hispanic Black women had modestly lower adjusted odds of LCS than non-Hispanic White women in the whole-cohort analysis; in the smaller pack-year subgroups the point estimates were similar in direction but no longer statistically significant. Hospital site and number of BCS visits were also independently associated with LCS uptake. These findings establish a baseline prior to implementation of the Pink & Pearl program at these sites and identify documentation completeness and tobacco treatment engagement as central correlates of LCS uptake in this population.

The racial disparity in LCS uptake observed in the whole-cohort model was no longer statistically significant in subgroup analyses restricted to women with documented pack-years. Point estimates in these subgroups remained below the null (AOR 0.65 among current smokers and 0.77 among former smokers), but confidence intervals were wide, reflecting sample sizes of 627 and 394 compared with 9,678 in the whole cohort. These data therefore cannot distinguish whether differential completeness of smoking history documentation contributes to the disparity or whether the subgroup analyses were simply underpowered. This remains a hypothesis for future work. It is a plausible one: incomplete smoking history documentation in EHRs is known to obscure LCS eligibility,^14^ and Black patients are less likely than White patients to receive clinician-initiated LCS recommendations.^15^

The association of pack-year documentation with LCS uptake may reflect greater clinical engagement: women who attend visits where structured tobacco history is collected are more likely to be identified as USPSTF-eligible and referred for LCS. The relationship may also run in the opposite direction, since ordering LDCT typically prompts documentation of pack-years, and our cross-sectional data cannot separate these pathways. The magnitude of the missingness, 63% of the analytic cohort lacking recorded pack-years and 79% lacking quit dates, is itself a finding. Strict application of USPSTF criteria in this population would have excluded most women because their eligibility could not be determined. This documentation gap represents a major barrier to implementing LCS programs in mammography settings. Embedding structured tobacco history intake into the BCS visit is one mechanism for closing this documentation gap, consistent with emerging evidence that mammography-coordinated outreach can increase LCS uptake among dual-eligible women,^16,17^ and our baseline estimates provide the comparator against which future evaluations of such strategies can be assessed.

Tobacco cessation medication use was independently associated with higher odds of LCS uptake across the whole cohort and the subgroups. This reflects two overlapping mechanisms. One, women receiving pharmacologic cessation support are more visible to the health system, and two, the prescribing encounter itself may create a clinical opportunity for LCS discussion and referral.

Current smoking status was similarly associated with higher uptake in the whole cohort, consistent with EHR-based work showing that current smokers are more likely than former smokers to receive LCS orders.^18^ This reflects a pattern in which active smokers are more readily identified as candidates for screening while former smokers, whose risk persists, may be less prominent in clinical documentation. This study has several strengths. We had a large cohort of women from two hospitals within a large academic health system and included nearly equal proportions of Black and White women, providing greater racial representation than other published LCS cohorts. Smoking history, screening events, and medication use were obtained from documented EHR data rather than self-report, reducing the potential for recall and social desirability bias. Finally, our analysis accounted for several potential confounders by adjusting for key variables that were readily captured in the large dataset. Although we adjusted for participant demographic, clinical, and place-based characteristics, residual confounding from unmeasured factors remains possible.

Several limitations should be noted. First, this analysis relies on EHR data from two hospitals within a single health system, and the patterns observed may not generalize to other settings or to women who do not engage with BCS. Geographic variation in uptake within the catchment area was not examined here and is a direction for future work. Second, the high prevalence of missing pack-years and quit dates limits the precision with which USPSTF eligibility can be ascertained and is a finding that needs further exploration. We addressed it by including documentation status as a covariate in the whole-cohort model and through stratified subgroup analyses, but residual misclassification of eligibility is likely. Third, the small sample sizes of the Other racial groups limited statistical power to assess differences for subgroups other than non-Hispanic White and non-Hispanic Black. Fourth, this is a cross-sectional analysis of LCS uptake at a single point and does not capture adherence to annual rescreening, which remains low in real-world settings and is a separate dimension of LCS quality.^19^ Finally, the EHR does not capture clinician-level factors such as referral patterns, recommendation quality, or patient-clinician communication, which prior work has shown to be important determinants of LCS uptake.

In conclusion, we found that baseline LCS uptake in this academic health system was higher than national LCS rates. Documentation completeness, tobacco cessation medication use, and current smoking status were most strongly associated with LCS uptake. Lower uptake among non-Hispanic Black women persisted in direction across analyses, though subgroup estimates were imprecise. The high prevalence of missing smoking history documentation is itself a barrier to identifying eligible women and warrants attention in its own right.

## Figures and Tables

**Figure 1.**
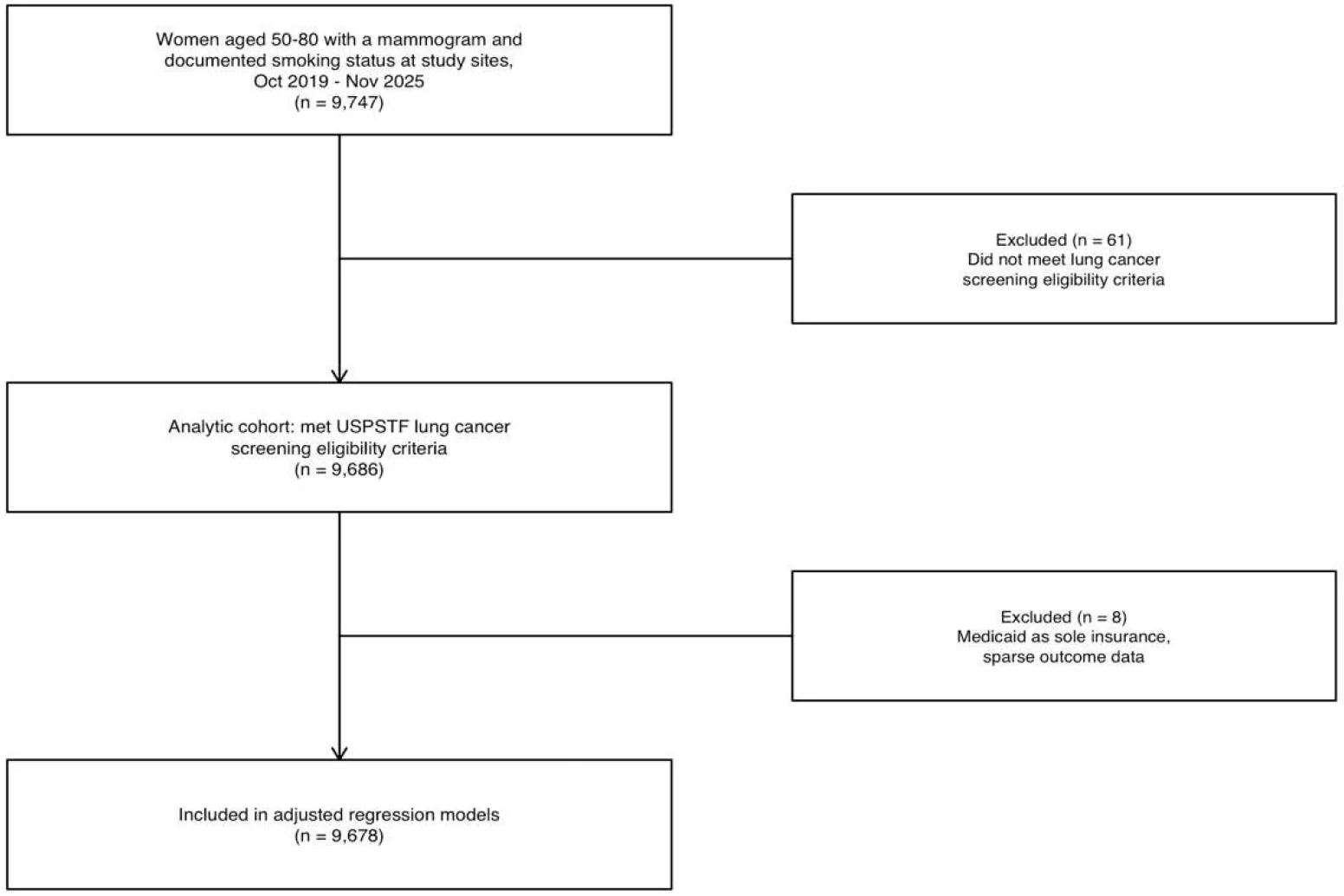
Flow diagram of cohort selection, Barnes-Jewish West County Hospital and Christian Hospital, October 2019 to November 2025.

**Table 1.** Baseline Characteristics of Women with Documented Smoking History by Screening Status (N = 9,686)

Characteristic	Overall N = 9,686^1^	Had LCS N = 3,209^1^	No LCS N = 6,477^1^
**Age (years)**	66 (59, 72)	67 (61, 73)	65 (58, 71)
**Age group**			
< 65	6,988 (72%)	2,195 (68%)	4,793 (74%)
>=65	2,698 (28%)	1,014 (32%)	1,684 (26%)
**Race/Ethnicity**			
Asian	48 (0.5%)	11 (0.3%)	37 (0.6%)
Hispanic	94 (1.0%)	28 (0.9%)	66 (1.0%)
Non-Hispanic Black	4,818 (50%)	1,601 (50%)	3,217 (50%)
Non-Hispanic White	4,625 (48%)	1,546 (48%)	3,079 (48%)
Other	101 (1.0%)	23 (0.7%)	78 (1.2%)
**Insurance type**			
Medicaid	8 (<0.1%)	5 (0.2%)	3 (<0.1%)
Medicare	3,945 (41%)	1,466 (46%)	2,479 (38%)
Multiple	2,624 (27%)	928 (29%)	1,696 (26%)
Private	3,109 (32%)	810 (25%)	2,299 (35%)
**Area Deprivation Index**			
1 (Least deprived)	601 (6.2%)	158 (4.9%)	443 (6.8%)
2	1,169 (12%)	333 (10%)	836 (13%)
3	1,728 (18%)	537 (17%)	1,191 (18%)
4 (Most deprived)	6,188 (64%)	2,181 (68%)	4,007 (62%)
**Smoking status**			
Current	2,212 (23%)	1,069 (33%)	1,143 (18%)
Former	7,474 (77%)	2,140 (67%)	5,334 (82%)
**Pack-years**	15 (5, 30)	25 (13, 40)	9 (3, 19)
**Pack-year data documented**			
Missing	6,084 (63%)	1,369 (43%)	4,715 (73%)
Documented	3,602 (37%)	1,840 (57%)	1,762 (27%)
**Quit status documented**			
Missing	7,649 (79%)	2,191 (68%)	5,458 (84%)
Documented	2,037 (21%)	1,018 (32%)	1,019 (16%)
**Tobacco cessation medication**	2,279 (24%)	1,208 (38%)	1,071 (17%)
**Any cancer history**	3,218 (33%)	1,302 (41%)	1,916 (30%)
**Hospital site**			
BJWCH	2,577 (27%)	743 (23%)	1,834 (28%)
CH	7,109 (73%)	2,466 (77%)	4,643 (72%)
**Alcohol use**			
Yes	4,053 (42%)	1,265 (39%)	2,788 (43%)
No	4,350 (45%)	1,619 (50%)	2,731 (42%)
Unknown	1,283 (13%)	325 (10%)	958 (15%)
**Number of breast cancer screenings**	5.0 (3.0, 7.0)	5.0 (3.0, 7.0)	5.0 (2.0, 7.0)

1 Median (Q1, Q3) for continuous; n (%) for categorical. Eligibility defined as women aged 50-80 with any documented smoking history (broader criteria).

**Table 2.** Factors Associated with LCS Uptake: Whole Cohort (N = 9,678)

	Unadjusted			Adjusted		
Characteristic	OR	95% CI	p-value	OR	95% CI	p-value
**Age group**						
< 65	—	—		—	—	
>=65	1.32	1.20, 1.45	<0.001	1.45	1.30, 1.62	<0.001
**Race/Ethnicity**						
Non-Hispanic White	—	—		—	—	
Asian	0.59	0.29, 1.13	0.13	0.75	0.34, 1.52	0.4
Hispanic	0.85	0.53, 1.31	0.5	1.29	0.78, 2.07	0.3
Non-Hispanic Black	0.99	0.91, 1.08	0.9	0.87	0.78, 0.98	0.018
Other	0.59	0.36, 0.92	0.027	0.81	0.47, 1.37	0.4
**Insurance type**						
Private	—	—		—	—	
Medicare	1.68	1.51, 1.86	<0.001	1.50	1.33, 1.70	<0.001
Multiple	1.55	1.39, 1.74	<0.001	1.44	1.27, 1.64	<0.001
**Area Deprivation Index**						
1 (Least deprived)	—	—		—	—	
2	1.11	0.89, 1.39	0.3	1.05	0.82, 1.34	0.7
3	1.26	1.02, 1.55	0.030	1.18	0.94, 1.50	0.2
4 (Most deprived)	1.53	1.27, 1.85	<0.001	1.43	1.14, 1.81	0.003
**Smoking status**						
Former	—	—		—	—	
Current	2.33	2.11, 2.57	<0.001	1.84	1.64, 2.07	<0.001
**Pack-year data documented**						
Missing	—	—		—	—	
Documented	3.59	3.28, 3.92	<0.001	2.90	2.63, 3.19	<0.001
**Tobacco cessation medication**						
No	—	—		—	—	
Yes	3.04	2.76, 3.35	<0.001	2.21	1.98, 2.48	<0.001
**Any cancer history**						
No	—	—		—	—	
Yes	1.63	1.49, 1.78	<0.001	1.61	1.45, 1.78	<0.001
**Hospital site**						
BJWCH	—	—		—	—	
CH	1.31	1.19, 1.44	<0.001	1.21	1.06, 1.39	0.005
**Alcohol use**						
No	—	—		—	—	
Yes	0.76	0.70, 0.84	<0.001	0.81	0.73, 0.89	<0.001
Unknown	0.57	0.50, 0.66	<0.001	0.77	0.66, 0.90	0.001
**Number of breast cancer screenings**	1.01	0.99, 1.02	0.3	1.04	1.03, 1.06	<0.001

Abbreviations: CI = Confidence Interval, OR = Odds Ratio. Eligibility: all women with any smoking history. Reference categories: Age < 65, Non-Hispanic White, Private insurance, ADI 1 (least deprived), Former smoker, Pack-year data missing, No cessation medication, No cancer history, BJWCH, No alcohol use. Eight Medicaid-only women excluded. Tests for effect modification of race by hospital site (LRT p = 0.26) and by area deprivation category (LRT p = 0.88) were not statistically significant.

**Table 3.** Factors Associated with LCS Uptake — Current Smokers, Pack-years ≥20 (N = 627)

	Unadjusted			Adjusted		
Characteristic^1^	OR^1^	95% CI^1^	p-value^1^	OR^1^	95% CI^1^	p-value^1^
**Age group**						
< 65	—	—		—	—	
>=65	0.92	0.60, 1.43	0.7	0.92	0.56, 1.51	0.7
**Race/Ethnicity**						
Non-Hispanic White	—	—		—	—	
Non-Hispanic Black	0.91	0.62, 1.33	0.6	0.65	0.40, 1.03	0.066
Other	0.52	0.13, 2.54	0.4	0.33	0.07, 1.86	0.2
**Insurance type**						
Private	—	—		—	—	
Medicare	1.46	0.95, 2.25	0.082	1.17	0.70, 1.93	0.6
Multiple	1.77	1.07, 2.97	0.027	1.72	0.99, 3.03	0.058
**Area Deprivation Index**						
1 (Least deprived)	—	—		—	—	
2	0.39	0.06, 1.67	0.3	0.23	0.03, 1.17	0.11
3	0.38	0.06, 1.48	0.2	0.23	0.03, 1.11	0.10
4 (Most deprived)	0.37	0.06, 1.29	0.2	0.23	0.03, 1.08	0.10
**Tobacco cessation medication**						
No	—	—		—	—	
Yes	1.63	1.12, 2.38	0.011	1.74	1.15, 2.65	**0.009**
**Any cancer history**						
No	—	—		—	—	
Yes	1.12	0.75, 1.68	0.6	1.24	0.79, 2.00	0.4
**Hospital site**						
BJWCH	—	—		—	—	
CH	1.11	0.66, 1.81	0.7	1.50	0.76, 2.91	0.2
**Alcohol use**						
No	—	—		—	—	
Yes	1.02	0.68, 1.54	>0.9	0.92	0.59, 1.44	0.7
Unknown	0.65	0.37, 1.15	0.13	0.77	0.41, 1.48	0.4
**Number of breast cancer screenings**	1.29	1.18, 1.42	<0.001	1.30	1.18, 1.43	**<0.001**

1 Asian, Hispanic, and Other race categories combined due to small cell sizes (n < 10 per group).

Abbreviations: CI = Confidence Interval, OR = Odds Ratio

Restricted to current smokers with pack-years ≥20. Smoking status excluded (all current smokers).

**Table 4.** Factors Associated with LCS Uptake — Former Smokers, Pack-years ≥20, Quit Date Documented (N = 394)

	Unadjusted			Adjusted		
Characteristic^1^	OR^1^	95% CI^1^	p-value^1^	OR^1^	95% CI^1^	p-value^1^
**Age group**						
< 65	—	—		—	—	
>=65	0.96	0.52, 1.82	0.9	1.18	0.61, 2.35	0.6
**Race/Ethnicity**						
Non-Hispanic White	—	—		—	—	
Non-Hispanic Black	1.06	0.60, 1.86	0.8	0.77	0.38, 1.52	0.5
Other	2.15	0.24, 283	0.6	1.81	0.19, 240	0.7
**Insurance type**						
Private	—	—		—	—	
Medicare	0.64	0.29, 1.33	0.2	0.81	0.35, 1.78	0.6
Multiple	0.62	0.27, 1.35	0.2	0.72	0.31, 1.63	0.4
**Area Deprivation Index**						
1 (Least deprived)	—	—		—	—	
2	1.46	0.23, 7.30	0.7	1.05	0.16, 5.49	>0.9
3	1.81	0.30, 8.19	0.5	1.27	0.20, 6.40	0.8
4 (Most deprived)	1.68	0.31, 6.19	0.5	0.83	0.13, 3.92	0.8
**Tobacco cessation medication**						
No	—	—		—	—	
Yes	1.38	0.78, 2.44	0.3	1.61	0.88, 2.96	0.12
**Any cancer history**						
No	—	—		—	—	
Yes	0.85	0.48, 1.49	0.6	1.04	0.57, 1.90	>0.9
**Hospital site**						
BJWCH	—	—		—	—	
CH	2.21	1.16, 4.10	0.017	2.90	1.23, 6.79	0.015
**Alcohol use**						
No	—	—		—	—	
Yes	1.24	0.69, 2.31	0.5	1.30	0.68, 2.56	0.4
Unknown	0.80	0.26, 3.17	0.7	1.13	0.32, 5.95	0.9
**Number of breast cancer screenings**	1.05	0.95, 1.18	0.4	1.05	0.94, 1.18	0.4

1 Estimates from Firth penalized logistic regression with profile likelihood 95% confidence intervals. The Other race category (n=6, all screened) exhibited complete separation; Firth penalization applied to all model coefficients for consistency. Smoking status excluded (all former smokers). Restricted to those with documented quit date.

Abbreviation: CI = Confidence Interval

## Data Availability

The data underlying this study contain protected health information from the Barnes-Jewish Healthcare electronic health record and cannot be shared publicly. The Institutional Review Board of Washington University approved this study under a waiver of informed consent, and the terms of that approval do not permit release of individual-level data. Aggregated data and analytic code are available from the corresponding author on reasonable request and subject to institutional approval.
